# Abdominal vascular syndromes: characteristic imaging findings*

**DOI:** 10.1590/0100-3984.2015.0136

**Published:** 2016

**Authors:** Leandro Cardarelli-Leite, Fernanda Garozzo Velloni, Priscila Silveira Salvadori, Marcelo Delboni Lemos, Giuseppe D'Ippolito

**Affiliations:** 1 MD, Radiologist in the Department of Diagnostic Imaging of the Escola Paulista de Medicina da Universidade Federal de São Paulo (EPM-Unifesp), São Paulo, SP, Brazil.; 2 Tenured Associate Professor in the Department of Diagnostic Imaging of the Escola Paulista de Medicina da Universidade Federal de São Paulo (EPM-Unifesp), São Paulo, SP, Brazil.

**Keywords:** Gastrointestinal tract, Venous thrombosis, Arteriovenous fistula, Hemangioma, Trato gastrintestinal, Trombose venosa, Fístula arteriovenosa, Hemangioma

## Abstract

Abdominal vascular syndromes are rare diseases. Although such syndromes vary widely in terms of symptoms and etiologies, certain imaging findings are characteristic. Depending on their etiology, they can be categorized as congenital-including blue rubber bleb nevus syndrome, Klippel-Trenaunay syndrome, and hereditary hemorrhagic telangiectasia (Rendu-Osler-Weber syndrome)-or compressive-including "nutcracker" syndrome, median arcuate ligament syndrome, Cockett syndrome (also known as May-Thurner syndrome), and superior mesenteric artery syndrome. In this article, we aimed to illustrate imaging findings that are characteristic of these syndromes, through studies conducted at our institution, as well as to perform a brief review of the literature on this topic.

## Abstract (pt)

As síndromes vasculares abdominais englobam doenças raras, com sintomas e causas diversas, mas com achados de imagem característicos. Dividem-se em dois grupos distintos, a depender de sua origem, que pode ser congênita (síndrome de blue rubber bleb nevus, síndrome de Klippel-Trenaunay e telangiectasia hereditária hemorrágica/Rendu-Osler-Weber) ou compressiva (síndrome do "quebra-nozes", síndrome do ligamento arqueado, síndrome de Cockett ou May-Thurner e síndrome da artéria mesentérica superior). Neste artigo, o nosso objetivo é ilustrar achados de imagens determinantes dessas doenças, por meio de estudos adquiridos em nossa instituição, associando uma breve revisão da literatura.

## INTRODUCTION

The use of imaging methods to evaluate abdominal diseases has been discussed in a series of recent articles in the radiology literature of Brazil^(1-10)^. Abdominal vascular syndromes, although rare, are relevant because they can often represent a diagnostic challenge for the attending physician. Such syndromes have varied clinical presentations and distinct causes. Nevertheless, their imaging findings are characteristic and must be recognized by radiologists^(11)^.

Abdominal vascular syndromes can be divided in two major groups, according to their origin^(12)^: congenital or compressive. Congenital syndromes include several types of low-flow or high-flow vascular malformations, which can result in hemorrhagic complications. Compressive syndromes are caused by compression of the vasculature by adjacent anatomical structures or by compression of hollow visceral organs by vessels. Compressive syndromes lead to major hemodynamic alterations, such as ischemia and thrombosis, primarily in young, healthy patients^(12,13)^.

## CONGENITAL VASCULAR SYNDROMES

It is important for radiologists to identify imaging findings as part of a single syndrome, dispelling diagnostic doubts and contributing to treatment planning. The main symptom is bleeding, occult or overt, leading to anemia and consumption of coagulation factors in the most severe cases^(12)^. Congenital vascular syndromes can be classified as low-flow or high-flow. To differentiate between those two types, it is recommended that, in addition to a physical exam, Doppler ultrasound, magnetic resonance imaging (MRI), and angiography be employed^(12)^. Classifying the flow allows a more precise diagnostic assessment and contributes to defining the most appropriate treatment strategy^(12,14)^.

### Low-flow type

Low-flow congenital vascular syndromes predominantly originate from a malformation of the venous system and present multiple phleboliths as a common imaging characteristic. Among such syndromes, we describe the blue rubber bleb nevus and Klippel-Trenaunay syndromes.

**Blue rubber bleb nevus syndrome** - This syndrome is characterized by vascular malformations which mainly affect the skin and the gastrointestinal system. On the skin, there are typically dark blue lesions, with an elastic consistency, measuring up to 5.0 cm^(11)^.

**Figure 1:**
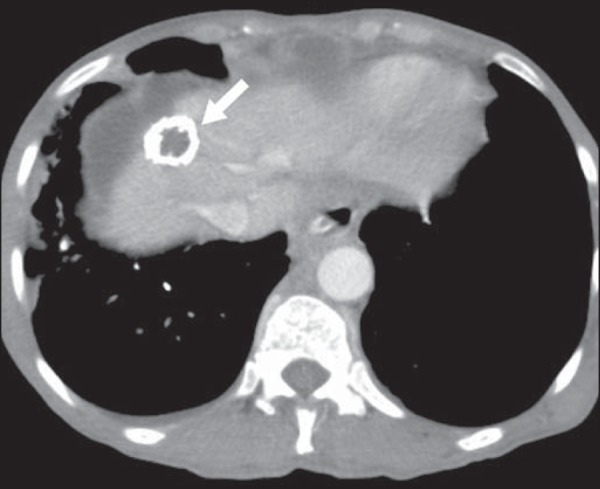
Blue rubber bleb nevus syndrome. Axial CT scan showing a calcified hepatic nodule (arrow), suggestive of hemangioma.

**Figure 2:**
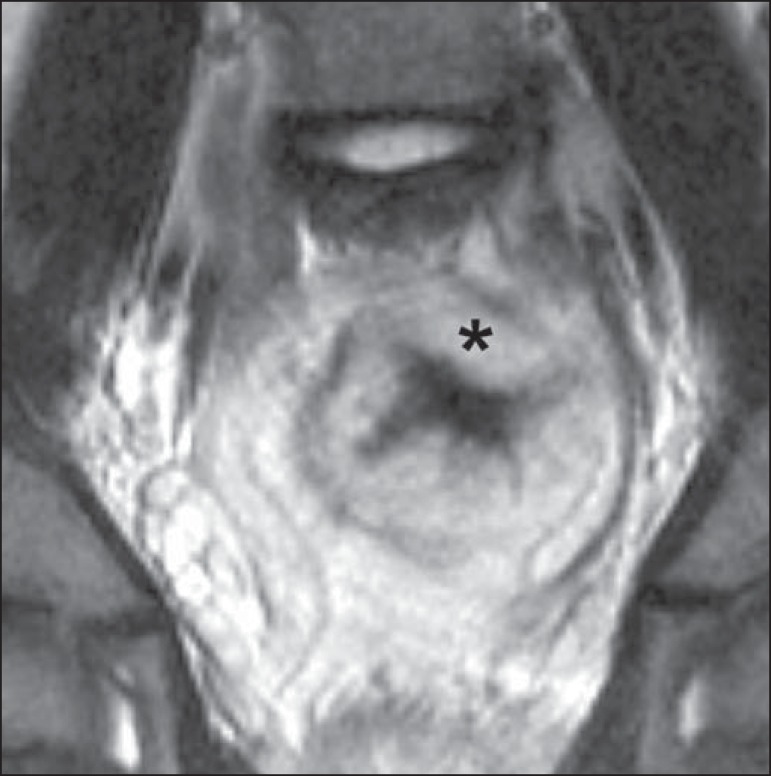
Blue rubber bleb nevus syndrome. Sagittal T2-weighted MRI scan showing circumferential thickening of the rectum (asterisk) with high signal intensity.

Barium radiography of the gastrointestinal tract shows polypoid filling defects. Computed tomography (CT) shows multiple phleboliths, representing cavernous hemangiomas, most commonly in the colon, rectum, and liver (Figure 1). On MRI, the venous malformations are well defined, with isointense signals on T1-weighted images, hyperintense signals on T2-weighted images, and progressive, homogenous uptake of intravenous paramagnetic contrast medium (Figure 2), from which the low-flow nature of the syndrome can be inferred^(11)^.

**Figure 3:**
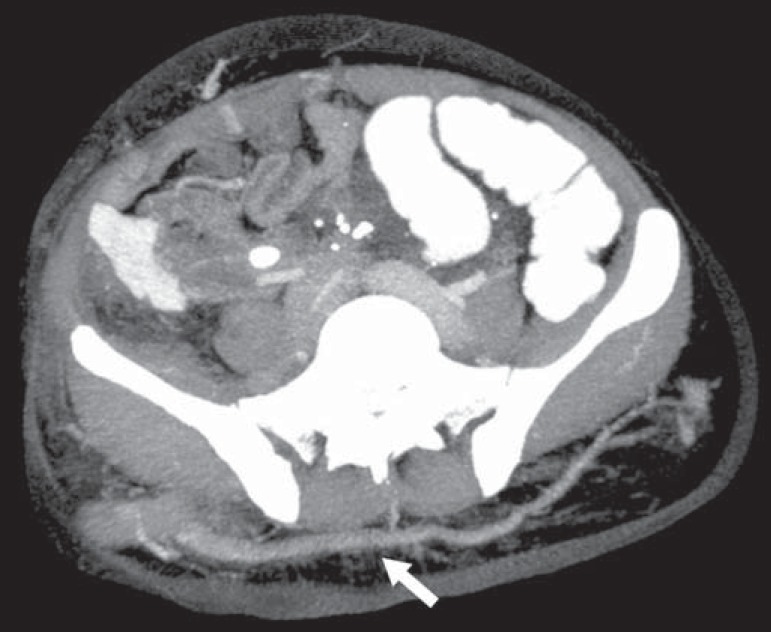
Klippel-Trenaunay syndrome. Axial maximum intensity projection CT reconstruction showing large caliber gluteal collateral circulation (arrow).

**Figure 4:**
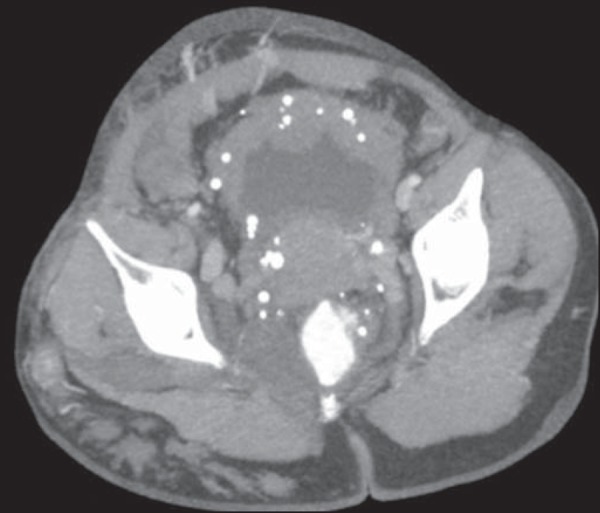
Klippel-Trenaunay syndrome. Axial maximum intensity projection CT reconstruction showing multiple phleboliths distributed throughout the bladder and rectum walls, which are thickened, indicative of cavernous hemangiomas.

**Klippel-Trenaunay syndrome** - A diagnosis of this syndrome, as manifested in a lower limb (Figure 3), can be made if at least two of the following criteria are met^(11)^: lateral giant varices; bone and soft tissue hypertrophy; and port-wine stains. In the gastrointestinal tract, Klippel-Trenaunay syndrome mainly affects the distal colon and rectum, although it can extend to the genitourinary structures in the pelvis. The main imaging findings, as depicted in Figure 4, include phleboliths, varices showing enhancement (in the later stages) after the use of intravenous contrast medium, and increased soft tissue mass in the lower limb^(11)^. The differential diagnoses include other diseases that cause gigantism of the limbs and vascular anomalies, such as the Proteus, Parkes-Weber, Bannayan-Riley-Ruvalcaba, and Maffucci syndromes, although only Klippel-Trenaunay syndrome causes venous malformations that present as "Port wine" stains^(15)^.

### High-flow type

High-flow congenital vascular syndromes are predominantly of arterial origin, the main example being hereditary hemorrhagic telangiectasia, also known as Rendu-Osler-Weber syndrome^(16)^.

The diagnostic criteria for high-flow congenital vascular syndromes include recurrent episodes of epistaxis; multiple vascular dilatations on the lip, palate, hands, or nose; arteriovenous malformations; and fistulas in organs such as the lungs, liver, brain, and bone marrow. Telangiectasia mainly involves the gastroduodenal mucosa and is generally limited, which hinders evaluation with radiological methods^(16)^.

**Figure 5:**
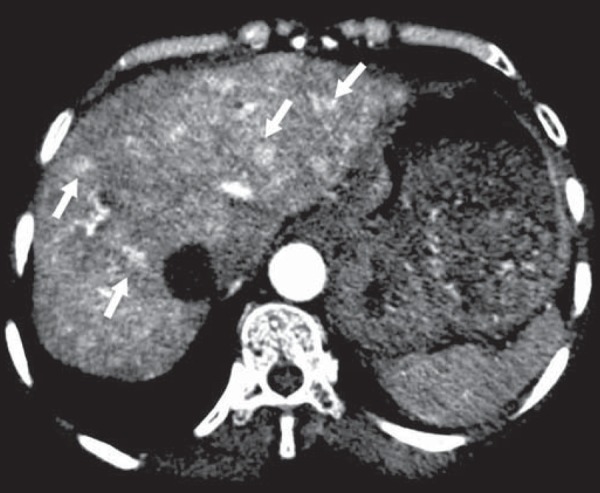
Rendu-Osler-Weber syndrome. Axial CT, in the arterial phase, with bone window setting for better visualization of multiple, small hypervascular perfusion disorders (arrows), resulting from intrahepatic shunts.

**Figure 6:**
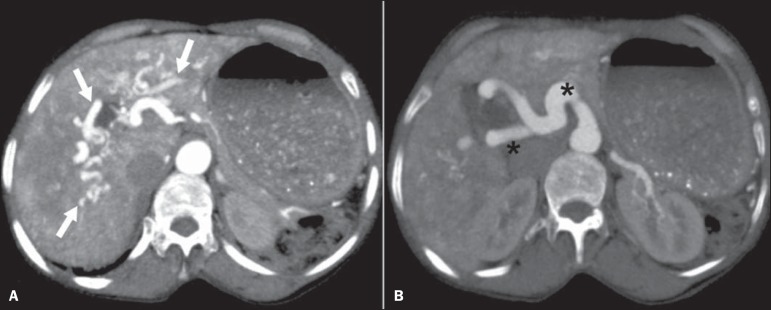
Rendu-Osler-Weber syndrome. Axial maximum intensity projection CT reconstructions. **A:** Ectasia and intrahepatic arterial sinuosity characteristic of arterial malformations (arrows). **B:** Accentuated increase in caliber throughout the hepatic artery and main branches (asterisk).

**Hereditary hemorrhagic telangiectasia (Rendu-Osler-Weber syndrome)** - In this disease, the main finding on imaging exams is hepatic involvement, which is present in up to 30% of patients. Such involvement manifests as large vascular bundles resulting from arteriovenous, arterioportal, or portovenous shunts (Figure 5). Those shunts can result in major clinical complications such as portal hypertension, ascites, hepatic encephalopathy, heart failure, and biliary necrosis^(16,17)^. Other findings include an increase in the caliber of the hepatic artery (> 1.0 cm) and telangiectasia, which is characterized by small hypervascular subcapsular nodules, less than 1.0 cm in diameter (Figure 6). It is noteworthy that the prevalence of focal nodular hyperplasia is 100 times higher in these patients with hereditary hemorrhagic telangiectasia than in the general population, information that can limit the number of unnecessary liver biopsies^(16,17)^.

## COMPRESSIVE VASCULAR SYNDROMES

In general, compressive vascular syndromes occur when the vascular structures in the abdomen and pelvis compress or are compressed by adjacent anatomical structures. Compression of the celiac trunk, left iliac vein, or renal vein is a common finding in the daily practice of radiology, a direct clinical correlation being indispensable when assigning weights to those radiological signs^(13)^. Compressive vascular syndromes manifest as nonspecific symptoms, such as pain in the upper abdomen and flanks, nausea, emesis, weight loss, and hematuria^(13)^, depending on the structure affected. Such syndromes include the so-called "nutcracker" syndrome, median arcuate ligament syndrome, Cockett syndrome or May-Thurner syndrome, and superior mesenteric artery syndrome.

**Nutcracker syndrome** - This syndrome involves compression of the left renal vein, typically between the aorta and the superior mesenteric artery, which impedes the drainage to the inferior vena cava, resulting in venous congestion or thrombosis. The syndrome can also occur in cases of a retroaortic renal vein that is compressed between the aorta and the vertebral body^(13)^.

Nutcracker syndrome primarily affects young and middle-age adults, with a slight predominance of females. The symptoms are the result of increased pressure in the left renal vein and include microscopic or macroscopic hematuria with a normocytic pattern, pain in the left flank, proteinuria, renal vein thrombosis, and pelvic varices^(18)^.

**Figure 7:**
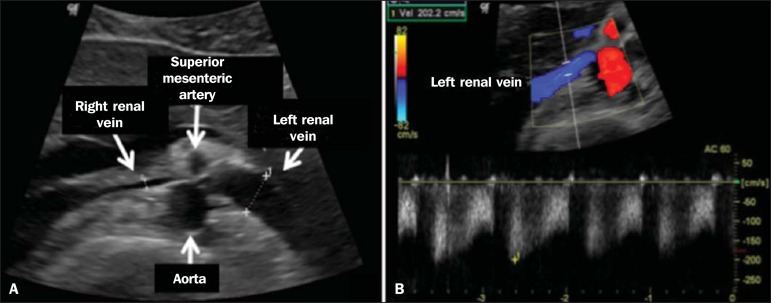
Nutcracker syndrome. **A:** B-mode ultrasound showing left renal artery stenosis between the superior mesenteric artery and the aorta, with pre-stenotic ectasia. **B:** Doppler ultrasound showing an accentuated increase in velocity in the left renal vein immediately downstream of the stenosis.

**Figure 8:**
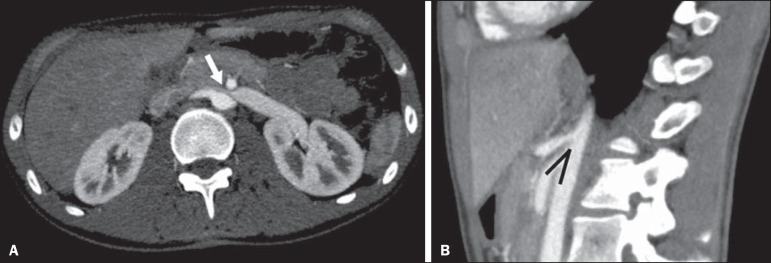
Nutcracker syndrome. **A:** Axial CT identifying stenosis in the left renal artery, between the superior mesenteric artery and the aorta (arrow), with pre-stenotic ectasia. **B:** Sagittal CT showing a reduction in the aortomesenteric angle (arrow point).

The initial imaging test for nutcracker syndrome is ultrasound, which is used in order to identify stenosis of the left renal vein where it crosses the superior mesenteric artery, with upstream dilatation, at a proportion of at least 3:1 (Figure 7). With spectral Doppler, it is possible to measure the post-stenotic peak velocity, which is normally above 100 cm/s^(18)^. As depicted in Figure 8, CT can show collaterals of the renal hilum, early opacification of the left gonadal vein (indicating reflux), and reduction of the aortomesenteric angle to less than 10°^(13)^. Such features can also be visualized on MRI scans.

**Figure 9:**
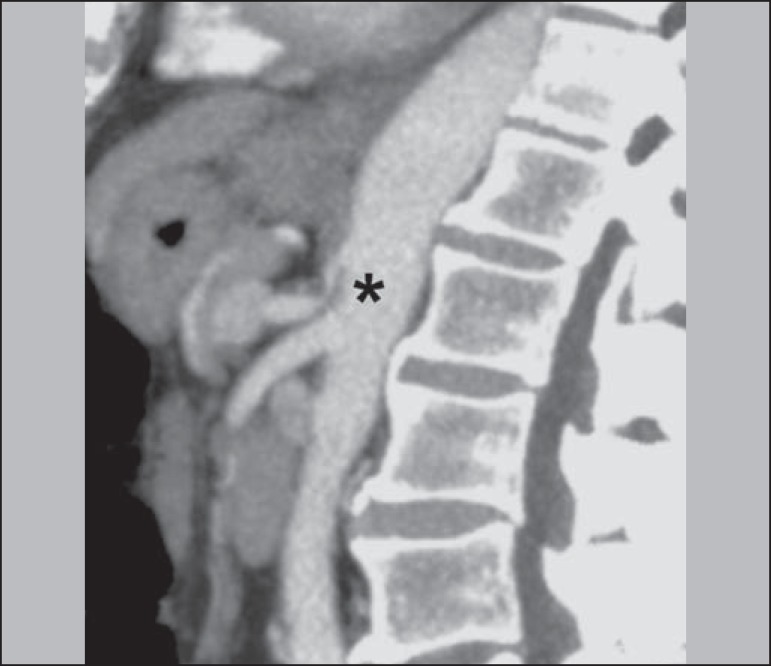
Median arcuate ligament syndrome. Sagittal CT showing a "hooked" aspect (trajectory flattening) of the celiac trunk (asterisk), with mild post-stenotic ectasia.

**Figure 10:**
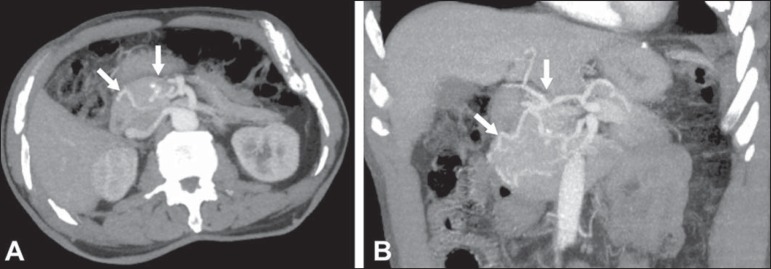
Median arcuate ligament syndrome. Axial **(A)** and coronal **(B)** maximum intensity projection CT reconstructions showing prominence (arrows) of the pancreaticoduodenal arcade (collateral circulation between the celiac trunk and the superior mesenteric artery).

**Median arcuate ligament syndrome** - The median arcuate ligament is a fibrous arch that connects the crura of the diaphragm near the aortic hiatus, at the level of the L1 vertebral body. It runs anterior to the aorta and superior to the celiac trunk. Compression of the proximal part of the celiac trunk by the median arcuate ligament can cause nonspecific symptoms such as epigastric pain and weight loss^(13)^. On CT and MR angiography, it is possible to observe focal stenosis and a "hook" aspect of the celiac trunk, which can be accentuated during expiration, with post-stenotic dilatation, as shown in Figure 9 ^(19)^. Median arcuate ligament syndrome is frequently associated with a prominence of the pancreaticoduodenal arcade, which is the collateral circulation between the celiac trunk and the superior mesenteric artery (Figure 10). The differential diagnosis with atherosclerosis is established by excluding foci of arterial calcification^(13)^.

**Figure 11:**
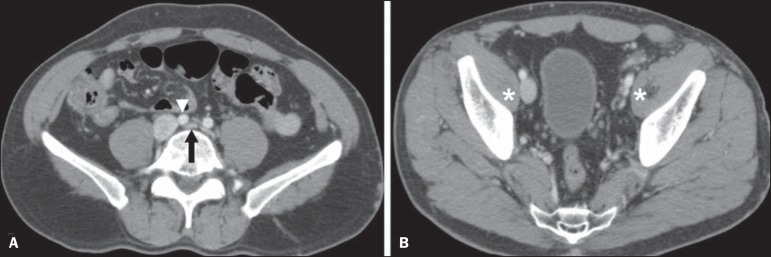
Cockett syndrome or May-Thurner syndrome. Axial CT. **A:** Compression of the left common iliac vein (arrow) by the right common iliac artery (arrowhead). **B:** Asymmetry of the external iliac veins (asterisk), with diffuse tapering on the left (chronic thrombosis) and ectasia on the right (resulting from increased pelvic collateral flow).

**Figure 12:**
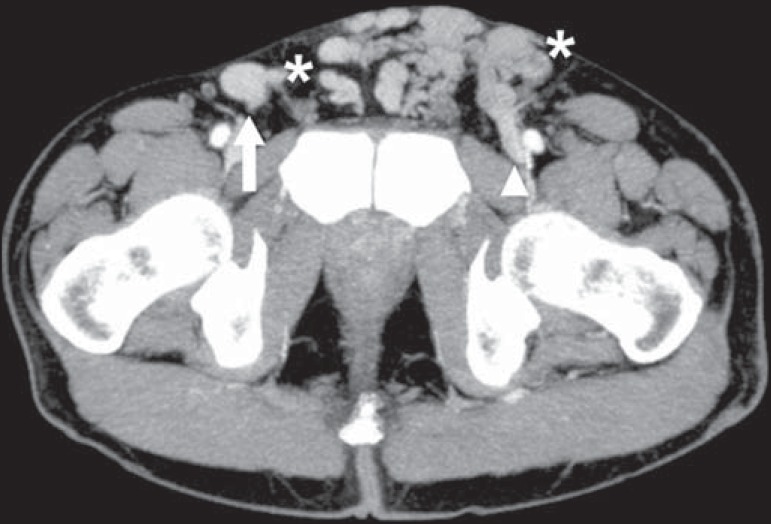
Cockett syndrome or May-Thurner syndrome. Axial CT showing suprapubic venous collateral circulation (asterisk), which creates communication between the right and left common femoral veins (arrow and arrowhead, respectively).

**Cockett syndrome or May-Thurner syndrome** - This condition involves compression of the left common iliac vein by the right common iliac artery^(20)^. Symptoms can be caused by the physical compression of the vein, between the artery and a vertebral body (extrinsic factor) or venous intimal hypertrophy, with the formation of fibrous beams due to the chronic contact with the artery (intrinsic factor), as depicted in Figure 11 ^(13)^. The syndrome mainly affects young and middle-aged adults, predominantly females. Symptoms can be acute, resulting from venous thrombosis, or chronic, caused by venous congestion, manifesting as edema of the left lower limb, pelvic varices, ulcers, pulmonary thromboembolism, and *phlegmasia cerulea dolens* (Figure 12)^(13)^.

**Figure 13:**
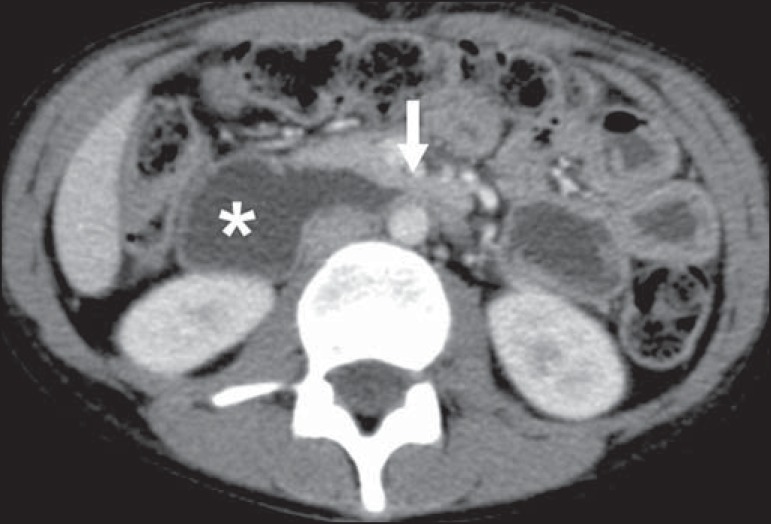
Superior mesenteric artery syndrome. Axial CT showing compression of the third part of the duodenum (arrow) between the superior mesenteric artery and the aorta, with consequent upstream dilatation (asterisk).

**Figure 14:**
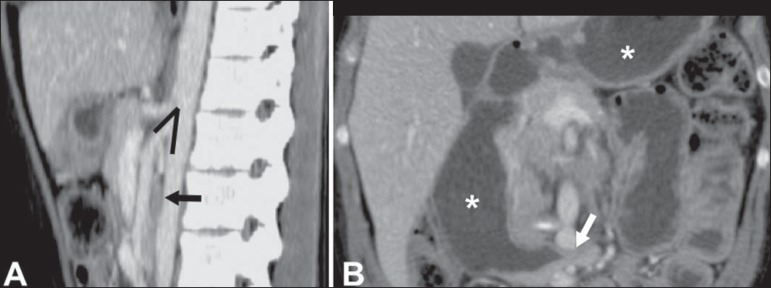
Superior mesenteric artery syndrome. **A:** Sagittal CT showing reduction of the aortomesenteric angle (arrow point) with reduction of the duodenal caliber at that level (arrow). **B:** Coronal CT showing gastroduodenal dilatation upstream of the compression.

**Superior mesenteric artery syndrome** - Also known as Wilkie's syndrome, this condition occurs when the third part of the duodenum is compressed between the superior mesenteric artery and the aorta (Figure 13). Under normal conditions, there is retroperitoneal fat around the third part of the duodenum, which avoids compression by creating an aortomesenteric angle ≥ 28º (Figure 14). Superior mesenteric artery syndrome can occur in patients who have experienced accentuated weight loss, who have undergone corrective surgery for scoliosis, or who present with anatomical variations in the ligament of Treitz, with consequent elevation of the duodenum. It mainly affects females between 10 and 39 years of age, causing postprandial upper abdominal pain that is relieved in ventral or left lateral decubitus, together with nausea, emesis, and weight loss^(13)^. The diagnosis should be suspected when the aortomesenteric angle is less than 25º and the aortomesenteric distance is less than 8 mm, in the presence of symptoms of duodenal obstruction^(13,21)^.

## CONCLUSION

For congenital vascular syndromes, the radiologist should be able to detect many of the malformations associated with the disease, as well as differentiating between the types of flow, evaluating the extent of the involvement, and determining the risk of complications. For the compressive syndromes, it is important to know their most common locations, recognizing that they can be incidental findings in asymptomatic patients. It should be borne in mind that the anatomical alterations detected should only be given weight when accompanied by the pertinent clinical correlation.

## References

[1] Nascimento JHR, Soder RB, Epifanio M (2015). Accuracy of computer-aided ultrasound as compared with magnetic
resonance imaging in the evaluation of nonalcoholic fatty liver disease in
obese and eutrophic adolescents. Radiol Bras.

[2] Vermelho MBF, Correia AS, Michailowsky TCA (2015). Abdominal alterations in disseminated paracoccidioidomycosis:
computed tomography findings. Radiol Bras.

[3] Szejnfeld D, Nunes TF, Fornazari VAV (2015). Transcatheter arterial embolization for unresectable symptomatic
giant hepatic hemangiomas: single-center experience using a lipiodol-ethanol
mixture. Radiol Bras.

[4] Bormann RL, Rocha EL, Kierszenbaum ML (2015). The role of gadoxetic acid as a paramagnetic contrast medium in
the characterization and detection of focal liver lesions: a
review. Radiol Bras.

[5] Barros RHO, Penachim TJ, Martins DL (2015). Multidetector computed tomography in the preoperative staging of
gastric adenocarcinoma. Radiol Bras.

[6] Rocha EL, Pedrassa BC, Bormann RL (2015). Abdominal tuberculosis: a radiological review with emphasis on
computed tomography and magnetic resonance imaging findings. Radiol Bras.

[7] Melo HJF, Goldman SM, Szejnfeld J (2014). Application of a protocol for magnetic resonance spectroscopy of
adrenal glands: an experiment with over 100 cases. Radiol Bras.

[8] Kierszenbaum ML, von Atzingen AC, Tiferes DA (2014). CT colonography: the value of this method in the view of
specialists. Radiol Bras.

[9] Herr K, Muglia VF, Koff WJ (2014). Imaging of the adrenal gland lesions. Radiol Bras.

[10] Cunha EFC, Rocha MS, Pereira FP (2014). Walled-off pancreatic necrosis and other current concepts in the
radiological assessment of acute pancreatitis. Radiol Bras.

[11] Elsayes KM, Menias CO, Dillman JR (2008). Vascular malformation and hemangiomatosis syndromes: spectrum of
imaging manifestations. AJR Am J Roentgenol.

[12] Nozaki T, Nosaka S, Miyazaki O (2013). Syndromes associated with vascular tumors and malformations: a
pictorial review. Radiographics.

[13] Lamba R, Tanner DT, Sekhon S (2014). Multidetector CT of vascular compression syndromes in the abdomen
and pelvis. Radiographics.

[14] Jackson IT, Carreño R, Potparic Z (1993). Hemangiomas, vascular malformations, and lymphovenous
malformations: classification and methods of treatment. Plast Reconstr Surg.

[15] Uller W, Fishman SJ, Alomari AI (2014). Overgrowth syndromes with complex vascular
anomalies. Semin Pediatr Surg.

[16] Carette MF, Nedelcu C, Tassart M (2009). Imaging of hereditary hemorrhagic telangiectasia. Cardiovasc Intervent Radiol.

[17] Agnollitto PM, Barreto ARF, Barbieri RFP (2013). Rendu-Osler- Weber syndrome: what radiologists should know.
Literature review and three cases report. Radiol Bras.

[18] Gulleroglu K, Gulleroglu B, Baskin E (2014). Nutcracker syndrome. World J Nephrol.

[19] Horton KM, Talamini MA, Fishman EK (2005). Median arcuate ligament syndrome: evaluation with CT
angiography. Radiographics.

[20] Mathur M, Cohen M, Bashir R (2014). May-Thurner syndrome. Circulation.

[21] Mearelli F, Degrassi F, Occhipinti AA (2014). Pinched: superior mesenteric artery syndrome. Am J Med.

